# Brightness and contrast adjustments influence proximal caries detection on handheld X-ray radiographs

**DOI:** 10.1590/1807-3107bor-2026.vol40.016

**Published:** 2026-03-30

**Authors:** Débora Costa RUIZ, Marcyele Natane da Silva MORAIS, Hugo GAÊTA-ARAUJO, Matheus Lima OLIVEIRA, Deborah Queiroz FREITAS, Francisco HAITER-NETO

**Affiliations:** (a) Universidade Estadual de Campinas – Unicamp, Piracicaba Dental School, Piracicaba, SP, Brazil; (b) Universidade de São Paulo – USP, Ribeirão Preto School of Dentistry, Department of Stomatology, Public Health, and Forensic Dentistry, Ribeirão Preto, SP, Brazil.

**Keywords:** Radiography, Dental, Digital, Dental Caries, Diagnostic Imaging, Radiographic Image Enhancement, X-Rays

## Abstract

This study evaluated the influence of brightness and contrast adjustments on the diagnosis of proximal caries lesions in radiographs acquired with a handheld X-ray device. A complementary metal oxide semiconductor sensor (SnapShot, Instrumentarium Imaging, Milwaukee, WI, USA) and a handheld Eagle X-ray device (Alliage, São Paulo, Brazil) were used to acquire radiographs of 20 mandibular molars and 20 mandibular premolars randomly arranged in 20 phantoms. The device settings were 60 kVp, 2.5 mA, and 0.45 seconds of exposure. Brightness and contrast of the resulting radiographs were modified in four combinations: a) −30% brightness and +30% contrast; b) −15% brightness and +15% contrast; c) +15% brightness and −15% contrast; and d) +30% brightness and −30% contrast. After randomization, the original radiographs and the four modified versions were individually assessed by five oral and maxillofacial radiologists to detect proximal caries lesions. The area under the receiver operating characteristic curve (AUC), sensitivity, and specificity were calculated from the examiners’ responses and compared with one-way analysis of variance (*p<*0.05). Intra- and inter-examiner agreement for radiographic diagnosis was assessed using the weighted kappa index. Sensitivity values for detecting proximal caries lesions on radiographs with increased brightness and decreased contrast (+ 30% brightness and −30% contrast) were significantly lower compared with the other combinations (p < 0.05), whereas AUC and specificity values were not influenced by the adjustments tested (p > 0.05). Therefore, increasing brightness and decreasing contrast on radiographs acquired with a handheld X-ray device is not recommended, since it may impair diagnostic accuracy for proximal caries lesions.

## Introduction

Handheld X-ray devices were introduced in the 1990s as substitutes for wall-mounted systems, primarily to support dental treatment for soldiers during military missions.^1,2^ Over time, these devices have been increasingly incorporated into everyday clinical settings. As their use expanded, concerns arose regarding safety and effectiveness, particularly the quality of radiographic images.^3,4^

These concerns are relevant to the development of guidelines on the use of handheld X-ray devices, since recommendations vary internationally. For instance, the European Academy of DentoMaxilloFacial Radiology adopts a more restrictive stance, whereas the American Dental Association imposes no restrictions provided that appropriate safety precautions are observed.^5,6^ Previous studies have investigated aspects of handheld X-ray use, such as aiming precision and potential image distortion in intraoral radiographs.^7,8^ Overall, the findings were promising, suggesting that these devices can produce reliable radiographs.^7,8^

In addition, research has demonstrated that handheld X-ray devices do not compromise diagnostic accuracy for detecting proximal caries lesions.^9,10^ No significant differences in diagnostic performance were found when comparing radiographs obtained using handheld devices with those acquired using wall-mounted devices.^9,10^ These results were consistent across studies employing both photostimulable phosphor plate (PSP) receptors and solid-state sensors, with neither receptor showing significant differences in accuracy between the two device types.^9,10^Nevertheless, objective assessments of image quality have indicated that radiographs obtained with handheld X-ray devices may exhibit variations in image characteristics.^11,12^ Depending on the device evaluated, studies have reported either increased brightness with reduced contrast^11^ or reduced brightness with increased contrast.^12^ These differences are likely due to variations in technical specifications and performance among the handheld X-ray models evaluated.

Brightness and contrast are relevant parameters that can be modified after radiographic acquisition to accommodate the observer’s preference.^13,14^ Such adjustments directly alter the gray values of a radiograph and may influence the perception of dental conditions.^13^ Considering earlier findings on the image quality of radiographs obtained with handheld X-ray devices, it is reasonable to hypothesize that adjusting brightness and contrast during post-processing could yield different diagnostic outcomes. Therefore, this study aimed to evaluate the influence of brightness and contrast adjustments on the diagnosis of proximal caries lesions in radiographs acquired with a handheld X-ray device.

## Methods

### Sample selection and preparation

After approval by the local research ethics committee (protocol number CAAE: 70610523.7.0000.5418), human premolars and molars with white spots or color changes indicative of proximal caries lesions were selected. Anomalous or restored teeth and teeth with cavities reaching the dentin were excluded. Based on these criteria, 40 teeth (20 premolars and 20 molars, totaling 80 proximal surfaces) composed the sample.

The gold standard for proximal caries lesions was confirmed with microcomputed tomography (SkyScan 1174, Bruker Corp., Kontich, Belgium), operated at 50 kVp, 800 µA, frame average of 1, 0.3˚ rotation step, 180˚ rotation, 15 µm voxel size, and a 0.5-mm-thick aluminum filter.^15,16^ The images were reconstructed with NRecon v.1.6.8 software (Bruker Corp., Kontich, Belgium), with 35% beam hardening correction, ring artifact correction of 5, and smoothing of 2.^15,16^ Two oral and maxillofacial radiologists, each with more than three years of experience, jointly examined the images in consensus, using DataViewer software (Bruker Corp., Kontich, Belgium). This evaluation classified 27 proximal surfaces as intact, 35 with enamel-limited caries lesions, and 18 with caries lesions extending to the dentin–enamel junction.

Each premolar was paired with a molar in random order, and the pairs were allocated to 20 silicone phantoms. Two non-test teeth were placed at the margins of each phantom to simulate proximal contact. A separate phantom containing four additional teeth was created to simulate the opposing dental arch.

### Radiographic acquisitions

The silicone phantoms were imaged with a size 1 complementary metal oxide semiconductor (CMOS) sensor (SnapShot, Instrumentarium Imaging, Milwaukee, USA) and an Eagle handheld X-ray device (Alliage, São Paulo, Brazil), operated at 60 kVp, 2.5 mA, and 0.45 seconds of exposure time. The device was fully charged before image acquisition. An acrylic locator ring was used to standardize exposure geometry, with a phantom–CMOS-sensor distance of 0.3 cm and focal-spot–CMOS-sensor distance of 40 cm, and a vertical angulation of 90°. In addition, an acrylic block was positioned between the X-ray device and the phantoms to simulate soft tissue attenuation.

The 20 resulting radiographs (one per phantom) were exported in Tagged Image File Format with 8-bit contrast resolution. Brightness and contrast were adjusted using PowerPoint (Microsoft Corporation, Redmond, USA) with four combinations: a) –30% brightness and +30% contrast; b) –15% brightness and +15% contrast; c) + 15% brightness and –15% contrast; and c) +30% brightness and –30% contrast. In total, 100 radiographs were produced, including the original images (Figure).

**Figure:**
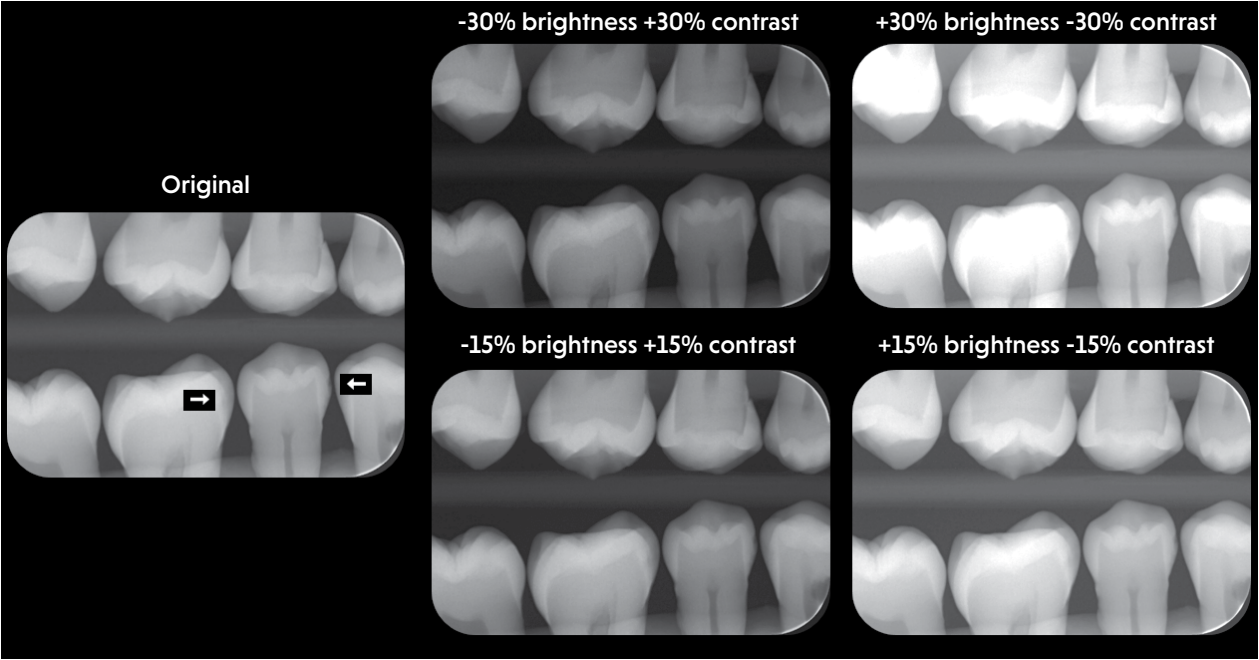
Original radiograph of a silicone phantom acquired with a handheld X-ray device and corresponding radiographs of the same phantom under four brightness and contrast combinations evaluated.

White arrows indicate proximal caries lesions.

### Radiographic evaluation

The presence of proximal caries lesions was evaluated by randomly arranging the 100 radiographs in a PowerPoint slideshow, one image per slide, without compression. Randomization was performed with an online random sequence generator (https://www.random.org). All radiographs were standardized to the same dimensions. Five oral and maxillofacial radiologists, blinded to the adjustments, independently graded the mesial and distal surfaces of each tooth on a 5-point scale: a) no proximal caries lesion; b) probable absence; c) uncertain; d) probable presence; e) presence of proximal caries lesion. Examinations were conducted in a silent room with low ambient lighting. Prior to assessment, practice images not included in the final sample were used to instruct the examiners.

Each examiner was advised to evaluate a maximum of 25 radiographs per day to avoid visual fatigue. Brightness and contrast modifications during analysis were not permitted. Interexaminer agreement was calculated based on these assessments. Thirty days later, 30% of the radiographs were selected, re-randomized, and re-evaluated by the same examiners for reproducibility analysis.

### Statistical analysis

Statistical analyses were performed with SPSS 25.0 software (SPSS, Chicago, USA) at α = 0.05. For each examiner and brightness/contrast combination, the area under the receiver operating characteristic curve (AUC), sensitivity, and specificity were calculated by comparing the examiners’ responses with the gold standard. Comparisons were conducted with one-way analysis of variance (ANOVA), followed by Tukey’s post hoc test to assess the effect of brightness and contrast adjustments. Intra- and inter-examiner agreement for proximal caries diagnosis was calculated with the weighted kappa index and interpreted as follows: 0.00–0.20, slight; 0.21–0.40, fair; 0.41–0.60, moderate; 0.61–0.80, substantial; and 0.81–1.00, almost perfect.^17^ The null hypothesis was that the brightness and contrast adjustments would not influence the detection of proximal caries lesions in radiographs acquired with a handheld X-ray device.

## Results

The values for AUC, sensitivity, and specificity are shown in Table 1. AUC values ranged from 0.67 to 0.75, with the original radiographs and three of the four tested combinations demonstrating acceptable discrimination (AUC > 0.7).^18^ Although the AUC was lower (0.67) for radiographs adjusted with the highest brightness and lowest contrast (+30 brightness and −30 contrast), this difference was not statistically significant compared with the other AUC values. Sensitivity values were low, ranging between 0.34 and 0.56, whereas specificity values were higher, ranging from 0.86 to 0.95. The highest brightness and lowest contrast adjustment (+30 brightness and −30 contrast) significantly reduced the observers’ ability to detect proximal caries lesions in radiographs acquired with a handheld X-ray device, with a statistically significant difference compared with the opposite adjustment (−30 brightness and +30 contrast) (p < 0.05)*.*

**Table 1:** Mean values (standard deviation) of the area under the receiver operating characteristic curve (AUC), sensitivity, and specificity according to brightness and contrast conditions tested.

Condition	AUC	Sensitivity	Specificity
Original	0.73 (0.05)	0.50 (0.09)	0.93 (0.05)
− 30 brightness and + 30 contrast	0.73 (0.07)	0.56 (0.14)	0.86 (0.11)
− 15 brightness and + 15 contrast	0.75 (0.06)	0.53 (0.10)	0.90 (0.07)
+ 15 brightness and − 15 contrast	0.73 (0.02)	0.45 (0.10)	0.94 (0.05)
+ 30 brightness and − 30 contrast	0.67 (0.02)	0.34 (0.10)*	0.95 (0.06)
p-value	0.194	0.035	0.321

*Significantly lower than − 30 brightness and + 30 contrast, according to one-way analysis of variance (p < 0.05).

Table 2 presents the intra- and inter-examiner agreement values. Intra-examiner agreement ranged from 0.594 to 0.852 (moderate to almost perfect), whereas inter-examiner agreement ranged from 0.301 to 0.620 (fair to moderate).

**Table 2:** Intra- and inter-examiner agreements for proximal caries lesion detection.

Examiner	1	2	3	4	5
1	0.661	0.342	0.524	0.488	0.451
2		0.594	0.373	0.301	0.318
3			0.682	0.620	0.584
4				0.746	0.616
5					0.852

Bold values represent intra-examiner agreement.

## Discussion

Brightness and contrast settings are among the most frequently applied post-processing tools in daily clinical practice, assisting in the interpretation of radiographs.^19^ Considering the widespread adoption of handheld X-ray devices in the past decade, it is essential to determine how brightness and contrast adjustments influence radiographic quality and diagnostic accuracy. Our results refuted the null hypothesis, since radiographs with increased brightness and decreased contrast impaired the diagnosis of proximal caries lesions. Therefore, these post-processing tools should be used cautiously.

Although studies evaluating radiographs acquired with a handheld X-ray device remain limited,^4,7-11,20^ the effects of brightness and contrast adjustments on radiographs obtained with traditional wall-mounted devices have been investigated.^13,21,22^These studies assessed different diagnostic tasks, including root resorption, caries lesions, and periapical lesions.^13,21,22^ Overall, the findings indicated that brightness and contrast adjustments did not significantly affect diagnostic performance for these conditions. However, an interesting observation was that examiners tended to prefer radiographs with lower brightness and higher contrast when diagnosing caries and periapical lesions.^13,22^

Interestingly, while previous studies found that brightness and contrast adjustments were largely examiner preferences and did not significantly affect diagnostic accuracy, the present study demonstrated that these adjustments influenced the visualization of proximal caries lesions. Given that both the present study and previous investigations applied similar ranges of brightness and contrast modifications, the discrepancy in results may be explained by the type of X-ray device used. A prior study showed that radiographs obtained with the same handheld X-ray device employed in this research inherently exhibit increased brightness and decreased contrast compared with images acquired using a wall-mounted unit.^11^ Consequently, the additional increase in brightness and reduction in contrast during post-processing in our study may have amplified these inherent grayscale differences, ultimately impairing diagnostic accuracy. In summary, one of the four post-processing adjustments tested (+30 brightness and –30 contrast) probably resulted in radiographs with even greater brightness and reduced contrast compared to those in previous studies that used wall-mounted X-ray devices. In contrast, the other post-processing adjustments tested did not negatively influence the radiographic diagnosis.

Enhancement filters are also post-processing tools that alter radiographs.^23^ These filters operate by adjusting gray values after algorithm application, indirectly modifying brightness and contrast.^24,25^ According to the literature, only one study has specifically evaluated the influence of enhancement filters on radiographs acquired with a handheld X-ray device.^26^ That investigation assessed 12 filters—six from the VistaScan system (Dürr Dental, Bietigheim-Bissingen, Germany) and six from the Digora Toto system (Soredex, Tuusula, Finland). The findings indicated that none of the tested filters affected diagnostic accuracy for detecting caries lesions.^26^ A plausible explanation is that, although these filters modify image brightness and contrast, the magnitude of these changes is relatively small and therefore insufficient to affect radiographic diagnosis.

In contrast, several studies have investigated enhancement filters in radiographs obtained with wall-mounted X-ray devices.^23-25,27,28^ These studies reported that the Fine filter in the VistaScan system and the Sharpen filter in the Digora system were promising tools for detecting proximal caries lesions.^27,28^Both filters enhance image sharpness and contrast, and when applied to wall-mounted device radiographs, this increase in contrast improved diagnostic performance. However, such improvement was not observed in the present study. This discrepancy may be attributed to the handheld X-ray device applied in our research, which produces radiographs with distinct inherent brightness and contrast characteristics, as well as to variations in the digital imaging systems evaluated.

This study evaluated a CMOS sensor from the Snapshot system. The spatial and contrast resolution of radiographs can vary depending on the digital system employed. In general, solid-state sensors such as CMOS provide higher spatial resolution but lower contrast resolution than PSP receptors.^29,30^ Spatial resolution refers to the ability of the imaging receptor to distinguish fine details, while contrast resolution relates to its ability to differentiate gray values.^30^ Although these resolutions are intrinsic to digital imaging systems, they can also be affected by factors such as X-ray attenuation.^30^ Thus, the effect of brightness and contrast settings on radiographs obtained with a PSP receptor may differ from those acquired with a CMOS sensor. While previous studies suggested that handheld devices combined with a PSP receptor did not impair caries detection, the application of different brightness and contrast adjustments under these conditions may alter diagnostic outcomes.^9,10^ Therefore, further research is needed to clarify how the adjustments tested in the present study interact with PSP receptors and handheld devices, and how these factors influence radiographic diagnosis.

The intra-examiner agreement in this study ranged from moderate to almost perfect, while the inter-examiner agreement ranged from fair to moderate. The inter-examiner results can be attributed to the sample composition, which included incipient caries lesions. This choice was intentional, as it simulated real clinical conditions and the diagnostic challenges of daily practice. In early disease stages, demineralization is minimal, making detection on bitewing radiographs more challenging. Additionally, the inter-examiner values observed here are consistent with those reported in previous studies focusing on incipient lesions.^31,32^ By contrast, the intra-examiner agreement values were slightly higher than those reported in earlier investigations, which may be explained by the structured calibration session conducted prior to the radiographic assessments. Although this study employed an *ex vivo* methodology, specific measures were implemented to minimize this limitation: an acrylic block was placed in front of the silicone phantoms to mimic the attenuation of soft tissues.

All radiographic acquisitions were performed with the handheld X-ray device fully charged. This approach was based on previous research demonstrating that tube voltage in handheld X-ray devices tends to decrease as battery charge diminishes, which may reduce radiographic quality.^33^ Furthermore, although brightness and contrast adjustments are often applied according to examiner preferences using dedicated imaging software, this study employed a protocol with four predefined adjustment combinations. Adjustments were made in PowerPoint, a procedure previously adopted in the literature and considered not to compromise methodological rigor.^13,22^ This protocol ensured standardized evaluations rather than individualized clinical adjustments, allowing a more reliable determination of whether brightness and contrast adjustments influence diagnostic accuracy.

The present findings provide valuable insights into handheld X-ray devices. However, additional research is warranted to better understand their performance. Further investigations should explore how handheld X-ray devices influence diagnostic accuracy for different tasks, such as detecting root resorptions and fractures. It would also be useful to assess performance under varying exposure parameters, including different exposure times. Such studies could clarify the potential benefits and limitations of handheld X-ray devices in diverse diagnostic scenarios.

## Conclusion

Brightness and contrast adjustments influenced the detection of proximal caries lesions in radiographs acquired with a handheld X-ray device. Specifically, increased brightness combined with decreased contrast impaired diagnostic performance. Therefore, clinicians should apply such adjustments cautiously.

## Data Availability

The datasets generated during and/or analyzed during the current study are available from the corresponding author on reasonable request.
